# Economics of planning electricity transmission considering environmental and health externalities

**DOI:** 10.1016/j.isci.2022.104815

**Published:** 2022-07-21

**Authors:** Bowen Yi, Shaohui Zhang, Ying Fan

**Affiliations:** 1 School of Economics & Management, Beihang University, Beijing 100191, China; 2 Laboratory for Low-carbon Intelligent Governance (LLIG), Beihang University, Beijing 100191, China; 3 International Institute for Applied Systems Analysis (IIASA), Schlossplatz 1, 2361 Laxenburg, Austria

**Keywords:** Health sciences, Energy policy, Electrical system, Energy sustainability, Economics

## Abstract

Long-distance electricity transmission can achieve environmental benefits through the transfer of air pollutants. However, current electricity transmission investment decisions do not take enough environmental factors into account. This study combines Greenhouse Gas–Air Pollution Interactions and Synergies model with power system planning to reveal how regional differences in environmental and health losses affect the allocation of electricity at the spatial level. Based on the analysis of inter-provincial electricity interconnection in China, we find that the regional differences in environmental and health external costs of power generation are significant. Considering external costs in investment decisions will largely improve the economy of long-distance inter-regional electricity transfer dominated by ultra-high voltage lines, thus replacing a portion of intra-regional electricity transfer dominated by high voltage lines. Meanwhile, the increases in local health losses in major electricity exporting provinces are not significant, which can alleviate the regional equity issues caused by pollutant transfer.

## Highlights

• Regional differences in environmental costs of power generation are significant
• Environmental costs can improve the economy of inter-regional electricity transfer
• Ultra-high voltage lines will play a key role in future power grids
• Increment in local health losses for major electricity exporters is not significant

## Graphical abstract

**Figure:**
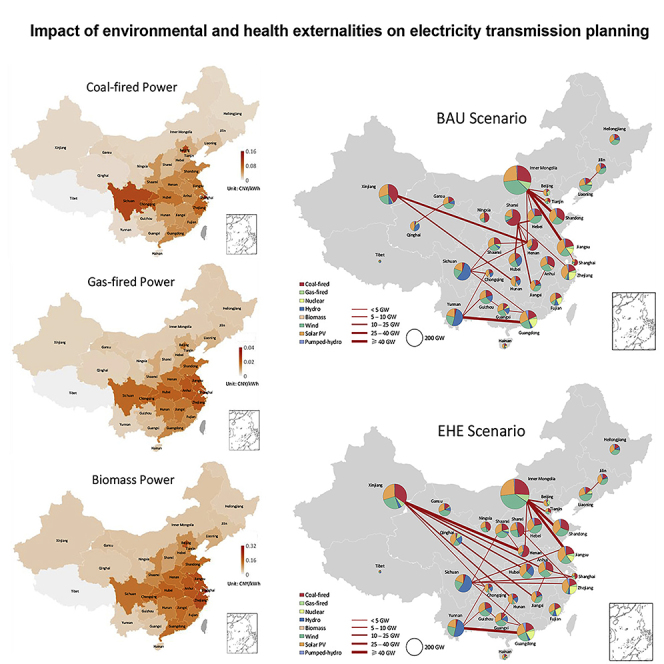

## Introduction

An important feature of electricity is that its generation may create a lot of pollution, but its consumption is completely clean. This feature allows long-distance electricity transmission to achieve environmental benefits through the transfer of pollutants (Li et al., 2021). In fact, the environmental benefits of electricity transmission are likely to be more than its contribution to carbon reduction. This is because the greenhouse effect is global, so the transfer of carbon emissions does not produce significant gains. Environmental pollution has obvious regional characteristics and mainly affects its surrounding areas. This means that the environmental impact of pollutant emissions is highly dependent on their geographical location, which makes it possible to reduce the overall impact by transferring emissions (Wilson and Hoehn, 2006). Therefore, environmental benefit is an important factor affecting the economy of planning electricity transmission.

However, environmental factors have not been given enough attention in electricity transmission investment decision making. Instead, the literature focuses more on the economic benefits (Voorspools and D’haeseleer, 2006), CO_2_ emissions reduction (Lindner et al., 2013), and the promotion of renewable energy (Abrell and Rausch, 2016). Even in some official reports on ultra-high voltage projects, environmental improvements are expressed only in terms of how much air pollutant emissions are reduced in electricity importing areas (Li et al., 2020), however, this expression has the following three limitations. First, this interconnection also leads to increased pollutant emissions in electricity exporting areas. Even if the purpose of electricity transfer is to absorb excess renewable energy, it still needs to be accompanied by thermal power to ensure stability and economy (He et al., 2020), which means that the emission factors on both sides of power import and export will affect the environmental benefits of electricity transfer, and this impact varies across time. Second, environmental benefits cannot be determined simply by pollutant emissions. For different areas, the same emissions may result in different concentrations, further leading to different human health losses (Xie et al., 2016). More importantly, air pollutants can also spread to other areas, causing indirect losses which are even higher than the direct impact on the local (Yi et al., 2020). Third, it is not enough to assess the environmental impact of built transmission lines. Only by incorporating environmental benefits into investment decisions can we find the future grid topology that maximizes overall social welfare, allowing us to evaluate the economy of planning electricity transmission more accurately.

There is extensive literature on the environmental impacts of power generation, most examples of which are based on air pollutant emissions, mainly nitrogen oxides (NO_X_), sulfur dioxide (SO_2_), primary particulate matter (PM_2.5_), and ammonia (NH_3_) (Alnatheer, 2006). Other studies have focused on the impact on human health of changes in PM_2.5_ concentrations caused by pollutant emissions, most of them using atmospheric chemistry transport models and exposure–response functions (Machol and Rizk, 2013). Because of China’s vast territory and large-scale inter-regional electricity transfers, it is of great significance to assess the differences in the environmental impact of power generation in different areas. In this field, Yi et al. (2020) evaluated the average environmental external costs of provincial electric power industry in 2010 and 2015. Wang et al. (2021) analyzed the environmental external costs of coal-fired power by province through an intake fractions (IFs) model.

Assessing the economy of long-distance electricity transmission is not a new topic (Van Der Weijde and Hobbs, 2012). Recent literature has introduced the scheduling of electricity on a micro temporal scale into the assessment to describe the utilization rate of transmission lines at different times and the intermittency of renewable energy (Perez et al., 2016). However, little research has taken environmental and health externalities into account. Some research took air pollutant emissions as investment constraints (Yi et al., 2016) whereas others were more concerned about the environmental impact after investment (Watcharejyothin and Shrestha, 2009). Existing studies fail to introduce environmental and health externalities into long-distance electricity transmission decision making. How regional differences in environmental losses affect the allocation of electricity at the spatial level is still not fully understood.

To address this gap, this study aims to evaluate the impact of environmental and health externalities on electricity transmission planning. The Greenhouse Gas–Air Pollution Interactions and Synergies (GAINS) model is adopted to assess the external costs of different types of power generation based on the provincial air pollution emission inventory in China’s electric power industry in 2018. It includes both direct local effects and indirect effects on other areas because of the diffusion of pollutants. Then, a large-scale Mixed Integer Linear Programming (MILP) model is constructed to characterize multi-regional co-optimization of power generation and transmission, and the external costs are introduced into the objective function of the MILP model. The target year of the model is chosen as 2035, meaning it needs to decide how to build new power grids on top of existing infrastructures to meet the additional demands from 2018 to 2035. Based on the analysis of inter-provincial electricity interconnection in China, we find that considering environmental factors will significantly improve the economy of long-distance inter-regional electricity transfer dominated by ultra-high voltage lines and reduce the total health losses at the national level. Meanwhile, increased local health losses in the major electricity exporting provinces are not particularly high, which alleviates the regional equity issues caused by pollutant transfer to a certain extent.

## Results

### External costs of power generation

We obtained the external costs of power generation in four major steps (see details in STAR Methods section): (1) Developing the provincial air pollution emission inventory in China’s electric power industry in 2018; (2) simulating the effects of different types of power generation on the spatial distribution of PM_2.5_ concentrations based on the GAINS model; (3) evaluating years of life lost through exposure-response functions and population data; and (4) calculating monetized health losses based on the value of a life year.

**Figure 1:**
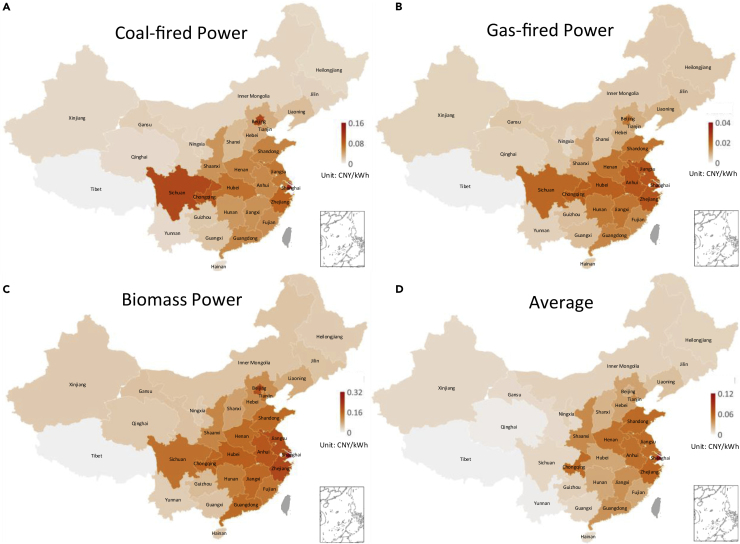
Provincial unit external costs of power generation The subfigures show the unit external costs of coal-fired power (A) gas-fired power (B) and biomass power (C) respectively. Subfigure (D) shows the provincial average external costs per kWh in 2018.

The external costs of different types of power generation technology vary greatly (see Figure 1). Coal-fired power accounts for the largest share in China’s power system, so its environmental externalities are of great concern. The external costs of coal-fired power range from 0.01 to 0.14 CNY per kWh, accounting for about 3–30% of the electricity generation costs in each province, whereas the external costs of gas-fired power are much smaller, about a third to a quarter of that of coal-fired power, depending on the region. In absolute amounts, these costs are generally lower than 0.03 CNY per kWh. Surprisingly, the external costs of biomass power are high, which has rarely been mentioned in previous literature.

From the spatial perspective, external costs vary greatly from region to region, which is quite different from the impact of carbon emissions, demonstrating the strong local nature of environmental pollution. There are many complex factors that can have an impact on external costs, including technology structures, fuel quality, population density, geographical location, atmospheric diffusion conditions, and economic levels. In the case of coal-fired power, the highest external costs are in Shanghai, Beijing, Sichuan and Chongqing. Among them, Shanghai and Beijing’s high costs are mainly because of economic factors and population density, whereas Sichuan and Chongqing’s are because of fuel quality and atmospheric diffusion conditions. Coal in Sichuan and Chongqing has a high sulfur content. This makes coal-fired power in these areas produce more SO_2_ emissions, which in turn form secondary PM_2.5_. Provincial emission factors of various air pollutants are shown in Figure S1. Sulfur and nitrogen content in coal is the main reason leading to the regional difference of emission factors of coal-fired power. Emission terminal control measures and energy efficiency also have an impact on emission factors, but depends on the specific air pollutant and power generation technology.

Nevertheless, the regional distribution of external costs for different types of power generation technology is similar, suggesting that fuel species mainly affect absolute levels of pollution but have limited impacts on regional differences. Almost all of China’s central and eastern provinces have relatively high external costs, including the Central power grid, East power grid, Guangdong of Southern power grid, Shaanxi of Northwest power grid, Liaoning of Northeast power grid, and North power grid excluding Inner Mongolia.

From the provincial average level, the regional distribution of external costs has changed again, which is closely related to the generation portfolio. For example, the average external costs in Sichuan, which is rich in hydropower, and Beijing, which is rich in gas-fired power, are not high. The areas with high average external costs are more concentrated in the coastal provinces from Shandong to Zhejiang and their neighbors, Henan and Anhui because of their coal-heavy generation mix, high population density, and well-developed economic levels.

### Comparison between emission sources and affected provinces

**Figure 2:**
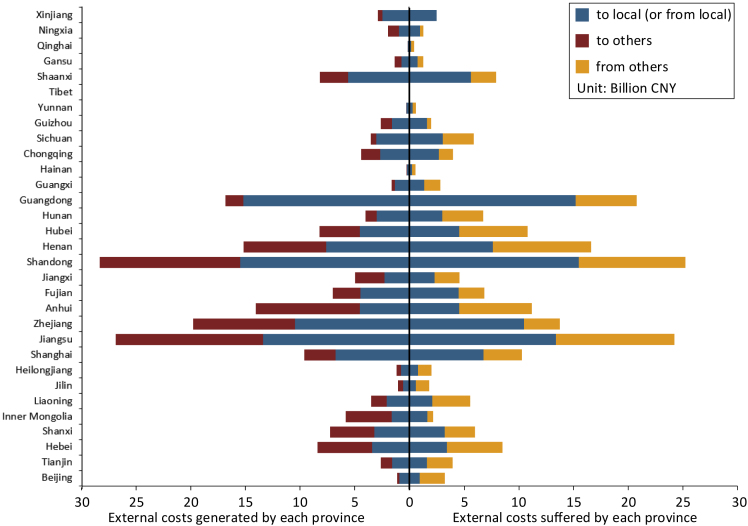
Total external costs generated or suffered by each province in 2018 The red bars represent the variable$G_{i}^{O}$in the Method section; the yellow bars represent the variable$S_{i}^{O}$; the blue bars represent the variables$G_{i}^{L}$and$S_{i}^{L}$, which are actually the same concept. The power structure composition of the external costs is shown in Figure S2.

Although air pollutant emissions have a strong local nature, they can still spread to other areas through atmospheric diffusion, especially to surrounding provinces. Figure 2 shows the total external costs generated or suffered by each province based on power generation data in 2018 (China Electricity Council, 2019). In most of the provinces with high external costs, the environmental impacts on other provinces are also high, and some even exceed the local impacts, which further illustrates the importance of considering the diffusion of pollutants to accurately assess external costs.

This phenomenon is most obvious in Anhui. Only 32.2% of the total external costs of its power generation are local impacts whereas the impacts on its surrounding Henan, Jiangsu, and Shandong provinces account for 14.5%, 13.1%, and 12.1%, respectively. All of these provinces are densely populated, and Shandong and Jiangsu also have high levels of economic development whereas Anhui has a relatively small population and modest economic situation. Therefore, although only a small part of the air pollutants produced by Anhui spread to other provinces, the health losses are still high. This further illustrates the importance of geographical location in assessing environmental externalities. It also shows that the cross influence between the central and eastern provinces of China has further raised their environmental external costs.

There are some provinces that do not generate a lot of health losses to others, but suffer significant pollution from surrounding provinces, such as Guangdong, Hubei, Hunan, Beijing, and Sichuan. This is related not only to atmospheric diffusion conditions but also to the characteristics of these provinces. In general, they tend to be more prone to health losses caused by air pollutants and their surrounding provinces have high levels of emissions. For example, only 28.2% of Beijing’s external costs related to power generation come from local sources whereas the impacts from Hebei are as high as 33.3%. This means that addressing environmental issues in certain provinces requires not only reducing local emission sources, but also considering actions in other provinces that have a significant impact on them.

### Optimal inter-provincial electricity transmission network

We evaluate the economy of electricity transmission planning by constructing a large-scale MILP model, which has the following three characteristics (see details in STAR Methods section): (1) It covers 31 provinces in China, and transmission lines can be built between any two provinces; (2) it is a co-optimization of power generation and transmission, including 8 types of power generation technologies and 2 types of power transmission technologies; and (3) it introduces the scheduling of electricity on a micro temporal scale (including 1,248 h) into macro investment decisions. We assume electricity demands at 2035 levels whereas existing power generation and transmission capacities are at 2018 levels and still not decommissioned in 2035. This means that the MILP model needs to decide how to build new power grids ($X_{i,j}^{v}$) to meet the additional demands from 2018 to 2035. The target year 2035 is chosen for two reasons. First, it is a key time point for the environmental goal of “Beautiful China” (Lu et al., 2020); second, the construction cycle of inter-regional power transmission lines is generally long, so its planning and layout should be advanced.

In order to investigate the effects of environmental and health externalities, two scenarios are considered in this study, namely business as usual (BAU) scenario and environmental and health externalities (EHE) scenario. Compared with the BAU scenario, we add environmental external cost ($Mh^{m}$) of thermal power (coal-fired power and gas-fired power) into the objective function of the EHE scenario. As for biomass power, although its health costs are high, it is politically unlikely to have environmental taxes imposed on it because agricultural wastes are more polluting if they are burned in scattered ways rather than used for power generation. China has six regional power grids, namely Northeast power grid, North power grid, Northwest power grid, East power grid, Central power grid, and Southern power grid. The first five grids are under the jurisdiction of State Grid Corporation of China, and the last one is under the jurisdiction of China Southern Power Grid. Inter-regional electricity transmission generally refers to the transfer involving different regional power grids whereas intra-regional electricity transmission as defined here refers to the transfer between provinces within a regional power grid.

**Figure 3:**
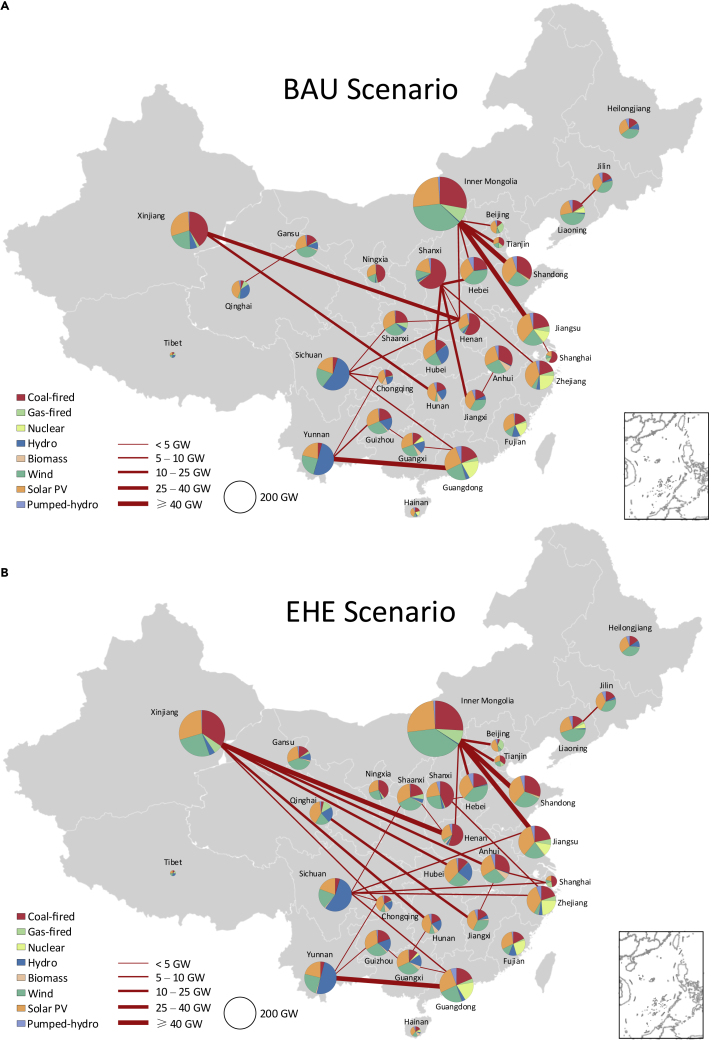
Provincial total capacity mix and new transmission lines under each scenario The subfigures show the results of BAU scenario (A) and EHE scenario (B) respectively. The area of the pie chart represents the amount of total installed capacity in 2035. The red lines show the newly built inter-provincial power transmission lines during 2018–2035, and the width of the lines represents the transmission capacity.

In the BAU scenario, the newly built transmission capacity is 343 GW, including 256 GW ultra-high voltage lines, and 87 GW high voltage lines (see Figure 3), which are cost-effective inter-provincial power grid plans that take into account regional resource endowments, intermittency of renewable energy, and grid topology. In the EHE scenario, the newly built transmission capacity is 398 GW, including 320 GW ultra-high voltage lines, and 78 GW high voltage lines. This means that when environmental and health externalities are considered, the number of cost-efficient transmission lines has increased, and the increase is mainly in ultra-high voltage lines. This is because economic level, population density, and electricity demand are often positively correlated, which makes electricity importing provinces have higher environmental external costs. Therefore, incorporating externalities into investment decisions will further widen the difference in power generation costs between the importing and exporting provinces. Moreover, provinces that are far away from each other tend to have significant differences in environmental external costs, and this part of electricity interconnection is often done through ultra-high voltage lines that are more efficient at transmission over long distances. High voltage lines, by contrast, tend to connect adjacent provinces within the same regional grid, with little difference in external costs between these provinces. In terms of electricity transmission volume in 2035, the total inter-provincial volume of the EHE scenario is 4,106.6 TWh, including 2,104.7 TWh for inter-regional transfer and 2,001.9 TWh for intra-regional transfer (see Figure S3). Compared with the BAU scenario, inter-regional transfer increases by 20.7% whereas intra-regional power transfer decreases by 4.9%, which further supports our findings.

From a provincial perspective, the impacts of environmental externalities on electricity exporters are relatively large, especially for Xinjiang and Shanxi. In the EHE scenario, electricity transfer from Xinjiang is greatly improved, and the number of newly built ultra-high voltage lines during the study period reaches 13, which is more than double that in the BAU scenario. These lines mainly connect central provinces, including Henan, Hunan, Hubei, Anhui, and Chongqing. In fact, Xinjiang is rich in coal, wind and solar resources, but because of its remote location, there is not a lot of electricity sent out at present. When we incorporate environmental factors into investment decisions, the cost advantage of power generation resources in Xinjiang will be further strengthened to offset the high transmission costs caused by distance. Meanwhile, the increased economy of electricity transmission also promotes the development of renewable energy in Xinjiang. Compared with BAU the capacity of onshore wind and solar PV under the EHE scenario increases by 91.7% and 53.5%, respectively.

In contrast, electricity transfer from Shanxi decreases significantly in the EHE scenario, and its connections with Hubei and Jiangxi are replaced by Xinjiang and Qinghai. Because of the close distance between Shanxi and the load center, its current power delivery is relatively large. But if environmental factors are considered, Shanxi will not be an economical electricity exporter in the future. On the one hand, Shanxi is close to heavily populated provinces such as Henan and Hebei, so the indirect effects of pollution diffusion make its external costs of power generation relatively high. On the other hand, the share of coal-fired power in Shanxi’s generation mix is high, which makes its power generation costs more susceptible to environmental tax. Inner Mongolia and Xinjiang, the two other major electricity exporters, have far more renewable resources than Shanxi. In addition, as for electricity importers, almost all provinces with high environmental external costs increase their electricity imports, mainly including Jiangsu, Zhejiang, Shanghai, Anhui, and Henan. Compared with the BAU scenario the net electricity imports of these provinces in the EHE scenario increase by 20.4%, 32.6%, 30.8%, 102.8%, and 13.3%, respectively.

### External benefits from inter-provincial electricity transfers

Electricity transfers from areas with low unit external costs to areas with high costs can reduce the total health losses at the national level. This external benefit is actually brought by the transfer of electricity rather than the cleanliness of the power structure. In the BAU scenario, this total external benefit is 73.403 billion CNY whereas it is increased to 82.260 billion CNY in the EHE scenario, indicating that considering environmental factors in investment decisions can make power generation more concentrated in areas with low health losses. It is worth noting that, electricity transfer does not necessarily lead to a reduction in air pollutant emissions (see Table S1), which further emphasizes the importance of characterizing the environmental effects through health losses rather than air pollution emissions. The provincial emissions of various air pollutants under each scenario are shown in Figure S4.

**Figure 4:**
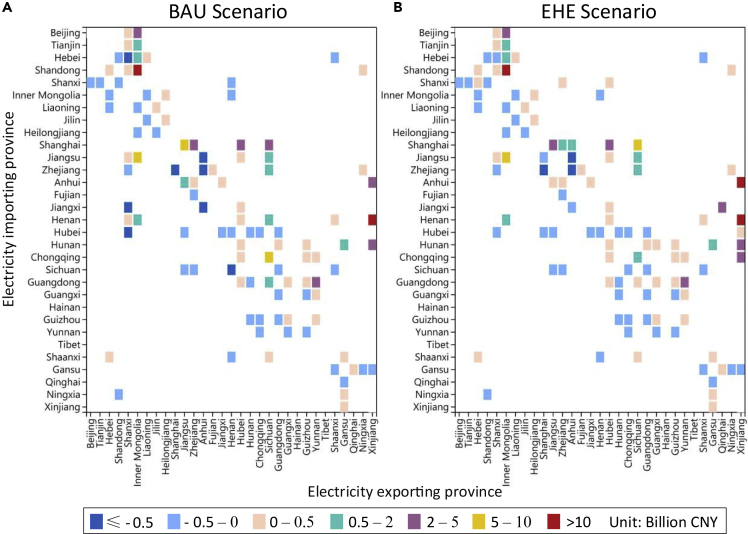
Province-specific external benefits from electricity transfers under each scenario The subfigures show the results of BAU scenario (A) and EHE scenario (B) respectively. The squares represent the transfer of electricity between each province in 2035, and the colors of the squares represent the range of external benefits of the transfer.

Figure 4 presents the province-specific external benefits from electricity transfers under each scenario. We can see that not all electricity transfers can bring environmental benefits, because external costs are only a small part of power transmission investment decisions. It is normal to transfer electricity from areas with high unit external costs to areas with low costs, but such transfer is generally not large in absolute terms. As we consider the transmission and dispatch of electricity at the hourly level, there may also be two-way transmission between some provincial pairs at different times because of the seasonality and intermittency of renewable energy, which inevitably leads to this phenomenon (see Figures S5 and S6). The external benefits of electricity transmitted from Inner Mongolia, Xinjiang, and Sichuan are the highest, as opposed to Shanxi and Anhui. Compared with the BAU scenario, the new cost-effective transmission lines in the EHE scenario are generally of high environmental benefits, such as Xinjiang – Anhui, Xinjiang – Chongqing, Qinghai – Jiangxi, and Sichuan – Shanghai. Meanwhile, the negative impacts of the lines connecting Shanxi with Hebei, Jiangxi, and Hubei are also reduced in the EHE scenario.

**Figure 5:**
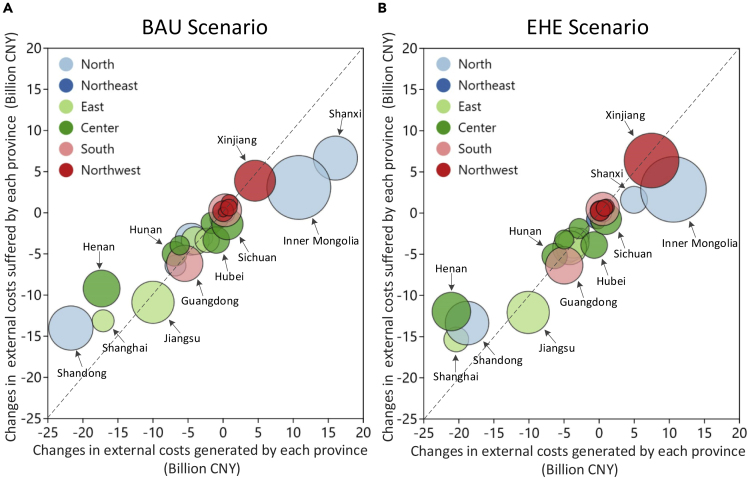
External cost changes caused by electricity transfers under each scenario The subfigures show the results of BAU scenario (A) and EHE scenario (B) respectively. The area of the pie chart represents the absolute amount of electricity transfers. North includes Beijing, Tianjin, Hebei, Shandong, Shanxi, Inner Mongolia; Northeast includes Heilongjiang, Jilin, Liaoning; East includes Jiangsu, Zhejiang, Shanghai, Anhui, Fujian; Center includes Henan, Hunan, Hubei, Jiangxi, Sichuan, Chongqing; South includes Guangdong, Guangxi, Hainan, Guizhou, Yunnan; Northwest includes Xinjiang, Tibet, Gansu, Qinghai, Ningxia, Shaanxi.

By collating the benefits of the lines in Figure 4 to the provincial level, we can obtain the changes in external costs caused by electricity transfers in each province (see Figure 5). These cost changes are generally negative for electricity importing provinces, meaning that the environmental impacts of power generation in these areas are reduced whereas the opposite is true for electricity exporting provinces. It can be seen that the increase in external costs because of electricity transfer in the major exporting provinces (Shanxi, Inner Mongolia, Xinjiang, Sichuan, Yunnan) is significantly lower than the reduction in the importing provinces (Shandong, Henan, Shanghai, Jiangsu), and this gap is further increased in the EHE scenario.

It is worth noting that, the external costs suffered by most provinces will be reduced because of electricity transfer, even in some electricity exporting provinces, such as Sichuan, Guizhou, and Fujian. This is because most of their surrounding areas are electricity importers, and the improvement of the environment in these surrounding areas brings indirect benefits to them. Provinces with large absolute transfers tend to experience greater changes in generation than they suffer, meaning that the local health losses in the major electricity exporting provinces are not particularly huge. This alleviates the regional equity issues caused by pollutant transfer to a certain extent. Provinces that suffer an increase in external costs include Northwest power grid, Inner Mongolia and Shanxi of North power grid, Heilongjiang and Jilin of Northeast power grid, and Yunnan of Southern power grid.

## Discussion

This study points out the importance of considering environmental and health externalities in electricity transmission planning. Compared with the generation costs, the environmental and health external costs of power generation cannot be ignored, and its regional differences are significant. Especially in the densely populated and economically developed central and eastern provinces of China, the cross influence caused by air pollutant diffusion further increases their external costs. This means that it is possible to reduce the total external costs at the national level by transferring pollutants over long distances through electricity interconnection rather than the cleanliness of the power structure.

This study also highlights the value of ultra-high voltage lines in China’s future electricity transmission network. Considering environmental and health external costs will significantly improve the economy of long-distance inter-regional electricity transfer dominated by ultra-high voltage lines, thus replacing a portion of intra-regional electricity transfer dominated by high voltage lines. The diffusion of air pollutants actually greatly reduces the environmental benefits of shifting electricity generation to nearby areas. For China’s major electricity exporting provinces, Sichuan and Yunnan have limited hydropower resources that will be almost fully developed by 2030. Therefore, in addition to Inner Mongolia, Xinjiang will play an increasingly important role in the future of electricity interconnection at the national level. When we incorporate environmental factors into investment decisions, the connections between Xinjiang and several central provinces will become cost-effective, thereby promoting renewable energy development and bringing significant environmental benefits.

The environmental benefits brought by electricity transfer are relatively scattered in spatial scale. The health losses suffered by most provinces could be reduced by the transfer of electricity at the national level. The provinces with increased health losses tend to be in remote areas. This means the impacts of air pollutant emissions in these provinces are mainly local. Meanwhile, the increases in local health losses in these provinces are not significant, which alleviates the regional equity issues caused by pollutant transfer to a certain extent. From the perspective of policy, the optimal spatial allocation of electricity resources can be achieved by levying environmental taxes on thermal power. However, environmental taxes must be different by regions, instead of adopting a national unified price or tax rate as has been done for CO_2_. These tax revenues should be used to compensate for health losses suffered by major electricity exporting areas.

Long-distance large-scale electricity transfers cannot only promote the development of renewable energy but can also bring additional environmental benefits. However, inter-regional electricity transactions in China still face local protectionism and regional trade barriers. Therefore, in addition to policies, mechanism design is also needed to solve the incentive issue, especially the design of inter-provincial and inter-regional electricity spot market.

### Limitations of the study

This study reveals the impacts of environmental and health externalities on electricity transmission planning. Nevertheless, there are several limitations to the present study and they deserve further exploration. First, limited by data availability and computing scale, this study selects the province as decision-making unit in the co-optimization of power generation and transmission. This ignores the electricity balance within the province and makes it difficult to characterize the specific location of electricity exporting in remote areas. Further improvement of the spatial scale is a difficult but meaningful research direction in the future. The increase in satellite data of various air pollutants will provide huge support for this field. Second, population mobility between regions not only causes changes in electricity demand, but also affects local health costs. According to Chen et al. (2020), over a relatively long temporal scale, population changes in China’s provinces are significantly different. The impact of population mobility on environmental assessments is therefore indispensable when long-term issues are involved. Third, soft-linkages between models need to be considered more in the future. For example, electricity demand is an exogenous parameter in this study, but it is actually affected by many uncertain factors, such as economic shocks. Soft-linkages with other models can help us identify the impact of these factors on the layout of electricity transmission.

## STAR★Methods

### Key resources table

**Table:** 

REAGENT or RESOURCE	SOURCE	IDENTIFIER
**Deposited data**		

Existing power generation and transmission structures	Compilation of Statistical Data of Electric Power Industry	https://cec.org.cn/detail/index.html?3-172623
Power generation and transmission costs	International Renewable Energy Agency; Yi et al. (2019a)	https://www.irena.org/publications/2019/Oct/Future-of-wind https://www.irena.org/publications/2019/Nov/Future-of-Solar-Photovoltaic https://www.iaee.org/energyjournal/article/3377
Load curve at hourly level	National Development and Reform Commission	http://www.gov.cn/xinwen/2019-12/30/content_5465088.htm
Capacity factor of renewable energy at hourly level	Pfenninger and Staffell (2016); Staffell and Pfenninger (2016)	https://doi.org/10.1016/j.energy.2016.08.060 https://doi.org/10.1016/j.energy.2016.08.068
Electricity demand prediction	China Energy & Electricity Outlook	https://news.bjx.com.cn/html/20201202/1119458.shtml
Power dispatch parameters	Yi et al. (2019a)	https://www.iaee.org/energyjournal/article/3377

**Software and algorithms**		

GAMS	GAMS Software GmbH	GAMS 25.1

### Resource availability

#### Lead contact

Further information and requests for resources should be directed to and will be fulfilled by the lead contact, Shaohui Zhang (s_zhang@buaa.edu.cn).

#### Materials availability

The study did not generate new materials.

### Method details

#### Air pollution emissions

The calculation of air pollution emissions is based on the GAINS model developed by the International Institute for Applied Systems Analysis (IIASA) (Amann et al., 2011). The GAINS model is widely used to determine air quality and health effects, which considers the location, technology, and fuel quality of power plants, the application rates of emission control measures, and their emission removal efficiencies (Qin et al., 2017). Furthermore, the International Energy Agency (IEA) World Energy Outlook 2019 (International Energy Agency, 2019) and the data of the China Energy Statistical Yearbook 2019 (National Bureau of Statistics, 2019a) were integrated into the GAINS model to develop the provincial air pollution emission inventory in China’s electric power industry in 2018. Four air pollutants related to PM_2.5_ are considered: SO_2_, NO_X_, primary PM_2.5_, and NH_3_.

#### Assessment of health impacts

Ambient PM_2.5_ concentrations are calculated on a 0.1° grid in the GAINS model following the methodology described by Amann et al. (2020). Atmospheric transfer coefficients are based on perturbation simulations with the global EMEP Chemistry Transport Model (Simpson et al., 2012) run at 0.5° × 0.5° resolution for the full meteorological year 2015, with separate reduction simulations for urban low-level sources done at 0.1° × 0.1° resolution. The province-weighted PM_2.5_ concentrations generated by GAINS are shown in Table S2, and a comparison with the observed data is shown in Figure S7. Population exposure is calculated by overlaying ambient PM_2.5_ with projected populations on the same grid, based on a fine resolution gridded population from the University of Southampton’s WorldPop data set. Provincial-level age-specific demographic data from the China Statistical Yearbook 2019 (National Bureau of Statistics, 2019b) are used for calibration.

This study only addresses the effect of PM_2.5_ concentrations on human health, although this may slightly underestimate health losses. Generally, PM_2.5_ is considered to be the most important cause of death among various air pollutants (Gao et al., 2018). Including all pollutant emissions into the accounting category may lead to an overestimation because of the correlation between the concentrations of various pollutants (Künzli, 2002). Premature deaths from total ambient PM_2.5_ by provinces and fuels in China’s electric power industry are calculated using the integrated exposure-response functions (IERs) employed by the WHO assessment on the disease burden from long-term exposure to ambient air pollution (World Health Organization, 2016), which relies on cause-specific mortality relative risk (RR) function and requires the application to a higher range of annual average concentrations in the study area. Referring to the Global Burden of Disease (GBD), we calculate premature mortality linked to four diseases in adults (aged 30 or older), namely chronic obstructive pulmonary disease (COPD), ischemic heart disease (IHD), stroke (STK), and lung cancer (LC), and for one illness among infants less than 5 years old, acute lower respiratory infection (LRI). The RR calculation for each disease is shown in Equation (1).

(Equation 1) $$RR_{a,k}\left(C\right)=\left\{ \begin{array}{l} 1+\alpha_{a,k}\left(1-e^{-\beta_{a,k}{\left(C-C_{0}\right)}^{\gamma_{a,k}}}\right),\;C\geq C_{0}\\ 1\;,\;C<C_{0} \end{array}\right.$$

where *C* represents annual PM_2.5_ concentration; *C*_*0*_ represents the minimum-risk concentration threshold; *a* and *k* represent age and disease group, respectively; *α*, *β*, and *γ* are the parameters of IER function taken from Apte et al. (2015).

Referring to Vienneau et al. (2015), the impact of PM_2.5_ exposure on population health loss is indicated by years of life lost (YLL), as shown in Equation (2).

(Equation 2) $$YLL=\sum_{a}\sum_{s}\left(\sum_{k}\left(\frac{RR_{a,k}\left(C\right)-1}{RR_{a,k}\left(C\right)}\cdot y_{a,s,k}\cdot POP_{a,s}\right)\cdot el_{a,s}\right)$$

where *s* represents sex group; *y* is the age-sex-specific mortality rate for disease *k* taken from the GBD study 2019; *POP* is the size of the exposed population; and *el* is the expectation of life at age *a* in *s*, which is from the World Health Organization annual life table.

Value of a life year (VOLY) is used to assess the monetary value of change in life expectancy. Thus, the monetized health losses (*HL*) are calculated as follows:

(Equation 3) HL=YLL⋅VOLY

Because of the absence of VOLY estimates in many areas, a common approach is to adjust health costs by per capita GDP based on a reference area, as shown in Equation (4). Holland (2014) recommends a European VOLY of 57,700 euros (in 2005 prices), which is adopted in this study. According to the GDP per capita in PPP and exchange rate, we calculate China’s average VOLY in 2018 at about 219,000 CNY. In fact, VOLY’s assessment is highly subjective. Desaigues et al. (2011) recommend a confidence interval of 25,000 euros to 100,000 euros based on a contingent valuation survey of nine European countries. Therefore, the VOLY used in this study is a moderate value.

(Equation 4) $$VOLY_{i}=VOLY_{ref}\cdot {\left(\frac{Q_{i}}{Q_{ref}}\right)}^{\theta }$$

where *ref* represents the reference region; *Q* is the per capita GDP; and θ is the income elasticity of health cost, which is set to 0.8 suggested by Maji et al. (2018).

Depending on the fuel, there are three types of power generation (*m*) that have environmental impacts, namely coal-fired power, gas-fired power, and biomass power. Other technologies considered in this study produce no air pollutants during power generation. Within each fuel category, there are also many specific technologies, but the differences between them are relatively low. We explore the environmental and health impacts of these three types of power generation in each province of China through a series of simulations based on the GAINS model. The *BASE* case includes all anthropogenic emission sectors and biogenic emissions. The $noPOW_{i}^{m}$ cases exclude emissions from all power plants belonging to fuel type *m* in province *i* whereas other settings are the same as the *BASE* case. The differences between *BASE* and $noPOW_{i}^{m}$ cases are defined as the external costs of power generation, as shown in Equation (5). A similar approach is used by Gao et al. (2018) to separate the contribution of various sectors to PM_2.5_ concentrations.

(Equation 5) $$B^{m}={\left[b_{i,j}^{m}\right]}_{n\times n}\;\Rightarrow \;b_{i,j}^{m}=\frac{\left(\frac{C_{j}^{BASE}-C_{j}^{noPOW_{i}^{m}}}{C_{j}^{BASE}}\right)\cdot HL\left(C_{j}^{BASE}\right)}{P_{i}^{m}}$$

where *i*, *j* represent the province, and i,j∈{1,2,3,...,n}; $C_{j}^{BASE}$ and $C_{j}^{noPOW_{i}^{m}}$ represent PM_2.5_ concentration in province *j* from the *BASE* and $noPOW_{i}^{m}$ cases simulations; $P_{i}^{m}$ denotes electricity generation; $B^{m}={\left[b_{i,j}^{m}\right]}_{n\times n}$ is defined as the external cost matrix of electricity generation, which refers to the health losses to province *j* for each unit of electricity generated by fuel type *m* in province *i*.

The main diagonal elements of matrix $B^{m}$ refer to the local health impact of electricity generation whereas the other elements refer to the impact on other provinces because of the diffusion of air pollutants. The unit external cost ($ec_{i}^{m}$) defined in this study includes the above two parts and is calculated as follows:

(Equation 6) $$ec_{i}^{m}=\sum_{j=1}^{n}b_{i,j}^{m}$$

#### Total external costs generated or suffered by each province

The matrix $B^{m}$ implies that the total external costs generated ($G_{i}$) and suffered ($S_{i}$) by each province are different, as shown in Equation (7). The external costs suffered by each province consist of two parts. The first is the impact from local power generation ($S_{i}^{L}$), and the second is the impact caused by the pollutant diffusion from other provinces’ power generation ($S_{i}^{O}$). Similarly, the external costs generated by each province include the local impact ($G_{i}^{L}$) and the impact on other provinces ($G_{i}^{O}$).

(Equation 7) $$\left\{ \begin{array}{l} G_{i}=G_{i}^{L}+G_{i}^{O}=\sum_{m}\left(b_{i,i}^{m}\cdot P_{i}^{m}\right)+\sum_{m}\sum_{j}^{j\neq i}\left(b_{i,j}^{m}\cdot P_{i}^{m}\right)\\ S_{i}=S_{i}^{L}+S_{i}^{O}=\sum_{m}\left(b_{i,i}^{m}\cdot P_{i}^{m}\right)+\sum_{m}\sum_{j}^{j\neq i}\left(b_{j,i}^{m}\cdot P_{j}^{m}\right) \end{array}\right.$$

#### Economic evaluation of electricity transmission planning

Levelized cost of electricity (LCOE) is often used to evaluate the economy of power generation investment (Tu et al., 2019). Similarly, we can construct an indicator to reflect the Levelized Benefit of Electricity Transmission (LBOET), as shown in Equation (8).

(Equation 8) $$LBOET=\frac{\sum_{t=1}^{L}\frac{\left(I_{j}^{t}+M_{j}^{t}-I_{i}^{t}-M_{i}^{t}-I_{i,j}^{t}-M_{i,j}^{t}\right)}{{\left(1+r\right)}^{t}}}{\sum_{t=1}^{L}\frac{T_{i,j}^{t}}{{\left(1+r\right)}^{t}}}$$

where *t* is the time period (generally years); *L* represents the lifetime; *r* is the discount rate; $T_{i,j}^{t}$ represents the amount of electricity transferred from province *i* to *j*; $I_{i}^{t},\;I_{i,j}^{t}$ represent the investment cost of power generation and power transmission, respectively; $M_{i}^{t},\;M_{i,j}^{t}$ represent the operation and maintenance (O&M) cost of power generation and power transmission, respectively.

If we assume that the investment cost only occurs at the beginning of the lifetime, and the O&M cost and transmission volume remain the same from one period to another, we can remove the time *t* from Equation (8) and simplify it to Equation (9).

(Equation 9) $$LBOET=\frac{\left(I_{j}-I_{i}-I_{i,j}\right)\cdot \tau +\left(M_{j}-M_{i}-M_{i,j}\right)}{T_{i,j}}$$

where τ represents the present value annuity conversion factor, which is equal to $\frac{r}{1-1/{\left(1+r\right)}^{L}}$.

However, this indicator is only suitable for evaluating the economy of a single line because of the neglect of the power grid topology (Xu et al., 2020). When the number of provinces is expanded from 2 to *n*, some lines will not be built even if their LBOETs are positive because there may be better alternatives. This issue can be translated into a co-optimization of power generation and transmission, that is, to find the optimal decision that makes the total system cost (*Z*) lowest while meeting the power demand (*D*) in each province, as shown in Equation (10).

(Equation 10) $$\begin{array}{l} \min\;Z=\sum_{i,j}^{n}\left(\left(I_{i}+I_{i,j}\right)\cdot \tau +\left(M_{i}+M_{i,j}\right)\right)\\ s.t.\;P_{i}+\sum_{j}^{j\neq i}T_{j,i}-\sum_{j}^{j\neq i}T_{i,j}=D_{i} \end{array}$$

With the increasing share of renewable energy, its intermittency makes the real-time balance of electricity more significant. This implies that macro investment decisions need to consider scheduling and balancing on micro temporal scales (typically hours) (Yi et al., 2019a). Therefore, this study uses the idea of power economic dispatch model to expand the variables *M*, *P*, and *T* in Equation (10), so that it can simulate the impact of real-time balance on power transmission investment on an hourly scale. The objective function of this co-optimization is extended into the form of Equation (11).

(Equation 11) $$\begin{array}{l} \min\;Z=\sum_{m,v}\sum_{i,j}^{n}\left(\left(I_{i}^{m}+I_{i.j}^{v}\right)\cdot \tau +\left(M_{i}^{m}+M_{i,j}^{v}\right)\right)\\ \;=\sum_{m,v}\sum_{i,j}^{n}\left(\left(I_{i}^{m}+I_{i.j}^{v}\right)\cdot \tau +\left(Mf_{i}^{m}+Mf_{i,j}^{v}\right)+\sum_{d,h}\left(Mv_{i,d,h}^{m}+Me_{i,d,h}^{m}+Ms_{i,d,h}^{m}+Mc_{i,d,h}^{m}+Mh_{i,d,h}^{m}\right)\right) \end{array}$$

where the superscripts *m* and *v* represent the different power generation technologies and power transmission technologies, respectively; the subscripts *d* and *h* represent typical day and hour, respectively; $Mf^{v}$ represents the fixed O&M cost of power transmission; $Mf^{m},Mv^{m},Me^{m},Ms^{m},Mc^{m},Mh^{m}$ represent the fixed O&M cost, variable O&M cost, energy cost, start-up cost, carbon cost, and environmental external cost of power generation, respectively. In particular, the environmental external cost is equal to the product of the unit external cost ($ec_{i}^{m}$) calculated by Equation (6) and the electricity generation ($P_{i,d,h}^{m}$).

The constraint conditions are shown in Equation (12). (I) shows the real-time matching of electricity supply and demand; (II) represents the dynamics of commitment states for thermal power; (III) is the maximum and minimum power output constraints for the committed capacities; (IV) represents the ramping up and ramping down constraints; (V) and (VI) limit the available power generation and transmission capacities on an hourly scale, respectively; (VII) is the reserve restraint; and (VIII) guarantees that the decision variables are non-negative.

(Equation 12) $$\;s.t.\left\{ \begin{array}{l} \left(\text{Ι}\right)\;\sum_{m}P_{i,d,h}^{m}+\sum_{v}\left(\sum_{j}^{j\neq i}\left(T_{j,i,d,h}^{v}\cdot \left(1-\Omega_{j,i}^{v}\right)\right)-\sum_{j}^{j\neq i}\left(T_{i,j,d,h}^{v}\right)\right)=D_{i,d,h}\\ \left(\text{ΙΙ}\right)\;U_{i,d,h-1}^{m}+US_{i,d,h}^{m}-UD_{i.d,h}^{m}=U_{i,d,h}^{m}\;\forall m\in Z\;\\ \left(\text{ΙΙΙ}\right)\;\xi^{m}\cdot U_{i,d,h}^{m}\leq P_{i,d,h}^{m}\leq U_{i,d,h}^{m}\;\forall m\in Z\;\\ \left(\text{ΙV}\right)\;-P_{i,d,h-1}^{m}\cdot \eta^{-}\leq P_{i,d,h}^{m}-P_{i,d,h-1}^{m}\leq P_{i,d,h-1}^{m}\cdot \eta^{+}\;\forall m\in Z\;\\ \left(\text{V}\right)\;U_{i,d,h}^{m}\leq f_{i,d,h}^{m}\cdot \left(CN_{i}^{m}+CO_{i}^{m}\right)\;\\ \left(\text{VΙ}\right)\;T_{i,j,d,h}^{v}\leq CN_{i,j}^{v}+CO_{i,j}^{v}\;\\ \left(\text{VΙΙ}\right)\;\sum_{m}\left(CN_{i}^{m}+CO_{i}^{m}-P_{i,d,h}^{m}\right)\geq D_{i,d,h}\cdot \lambda \;\\ \left(\text{VΙΙΙ}\right)\;CN_{i}^{m},CN_{i,j}^{v},U_{i,d,h}^{m},US_{i,d,h}^{m},UD_{i.d,h}^{m},P_{i,d,h}^{m},T_{i,j,d,h}^{v}\geq 0\; \end{array}\right.$$

where the set Z represents thermal power; $U^{m},US^{m},UD^{m}$ represent the committed capacity, start-up capacity, and shut-down capacity, respectively; $\Omega^{v}$ represents the transmission loss rate, which are proportional to the distance; $\xi^{m}$ denotes minimum power output rate; λ is the reserve rate; $\eta^{-},\eta^{+}$ represents maximum ramping down and ramping up rates, respectively; $CO^{m},CO^{v}$ represent the existing power generation and transmission capacities (see Tables S3 and S4), which are fixed parameters; $CN^{m},CN^{v}$ represent the newly built power generation and transmission capacities, which are decision variables; and $f^{m}$ is the capacity factor. For renewable energy, its capacity factor varies by hour.

For long-distance electricity transmission, the capacity of a single line is usually large, so the planning of transmission lines needs to adopt discrete variables, as shown in Equation (13). $X_{i,j}^{v}$ is a non-negative integer representing the number of newly built transmission lines. $bc^{v}$ denotes the standard capacity of each line. Therefore, this co-optimization is actually a MILP model.

(Equation 13) $$CN_{i,j}^{v}=X_{i,j}^{v}\cdot bc^{v}$$

#### External benefits from electricity transfers

The external benefits ($EB_{i,j}$) refer to the difference of the external cost per unit of electricity in the importing province and the exporting province multiplied by the transmission volumes, as shown in Equation (14). Through the optimization results we can calculate the external benefits of electricity transfers between any two provinces.

(Equation 14) $$EB_{i,j}=\left(\frac{Mh_{j}}{P_{j}}\cdot \left(1-\Omega_{i,j}\right)-\frac{Mh_{i}}{P_{i}}\right)\cdot T_{i,j}$$

Further, we divide the external benefits of each transmission pair into two parts. Electricity transfers increase the external costs of power generation for the exporting province whereas reducing the costs for the importing province. Thus, the provincial total external cost changes caused by electricity transfers can be calculated by summarizing the impacts of all transmission pairs, as shown in Equation (15). This includes two different perspectives, that is, changes in external costs generated ($ECC_{i}^{G}$) and suffered ($ECC_{i}^{S}$) by each province.

(Equation 15) $$\left\{ \begin{array}{l} ECC_{i}^{G}=\frac{Mh_{i}}{P_{i}}\cdot \sum_{j}^{j\neq i}\left(T_{i,j}-\left(1-\Omega_{j,i}\right)\cdot T_{j,i}\right)\\ ECC_{i}^{S}=\sum_{j}\left(ECC_{j}^{G}\cdot \frac{b_{j,i}}{ec_{j}}\right) \end{array}\right.$$

#### Model setting

At the spatial scale, this study includes 31 provinces (*i*) in China. Each province is a decision unit, and transmission line construction can take place between any two provinces. We assume that an optimal balance has been reached between cities within a province, meaning that electricity transfer within one province is beyond the scope of this study. At the temporal scale, one typical day is selected every week; that is, 52 typical days (*d*) are included. Further, each typical day consists of 24 one-hour periods (*h*), which means that the MILP model includes 1,248 time nodes. Provincial electricity demand projections at each time node are explained in Table S5.

This study includes 8 types of power generation technologies (*m*), namely coal-fired power, gas-fired power, nuclear power, hydropower, biomass power, onshore wind, solar PV, and pumped-hydro storage. Because of the long distance between different provinces, this study assumes only 2 types of power transmission technologies (*v*), namely ultra-high voltage line (±800 kV) and high voltage line (±500 kV) (Yi et al., 2019b). Kirchhoff’s current law is respected in the model whereas the voltage law is not included (He et al., 2016). This MILP model contains 998,677 variables and 1,269,501 equations, of which 1,860 variables are discrete. It is solved by the CPLEX algorithm based on GAMS software.

## Data Availability

• This paper analyzes existing, publicly available data. These accession numbers for the datasets are listed in the key resources table and Method details. • This paper does not report original code, which is available for academic purposes from the lead contact upon reasonable request. • Any additional information required to reanalyze the data reported in this paper is available from the lead contact on request.
