# Supplementary material for: Economics of planning electricity transmission considering environmental and health externalities
Source: iScience. 2022 Jul 21;25(8):104815. doi: 10.1016/j.isci.2022.104815 (PMC9385690; doi:10.1016/j.isci.2022.104815)
Supplement: Document S1. Figures S1–S7 and Tables S1–S5 [file mmc1.pdf]

iScience, Volume *25*

Supplemental information

Economics of planning electricity transmission considering environmental and health externalities

Bowen Yi, Shaohui Zhang, and Ying Fan

Supplemental Figures

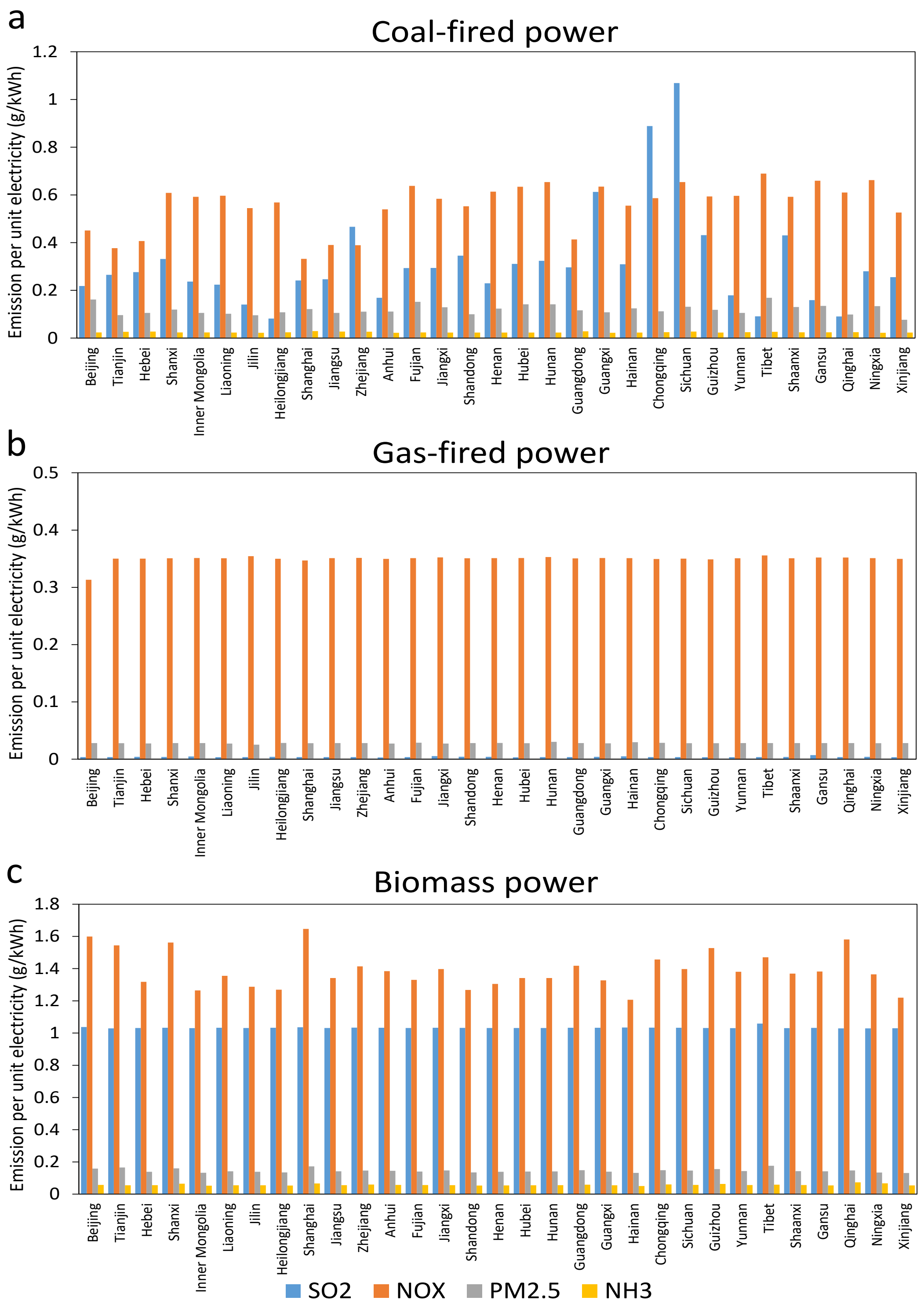

Figure S1. Provincial emission factors of various air pollutants in 2018, Related to Figure 1. The subfigures show the emission factors of coal-fired power (a), gas-fired power (b), and biomass power (c), respectively.

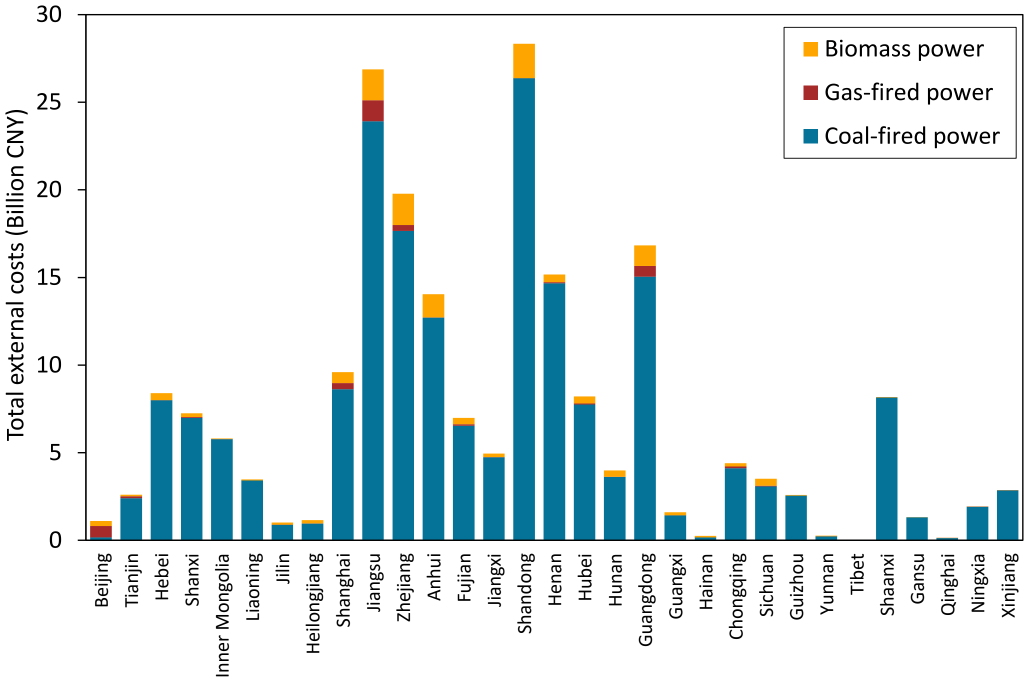

Figure S2. Total external costs by fuel type in 2018, Related to Figure 2.

It is based on the provincial power generation data in 2018.

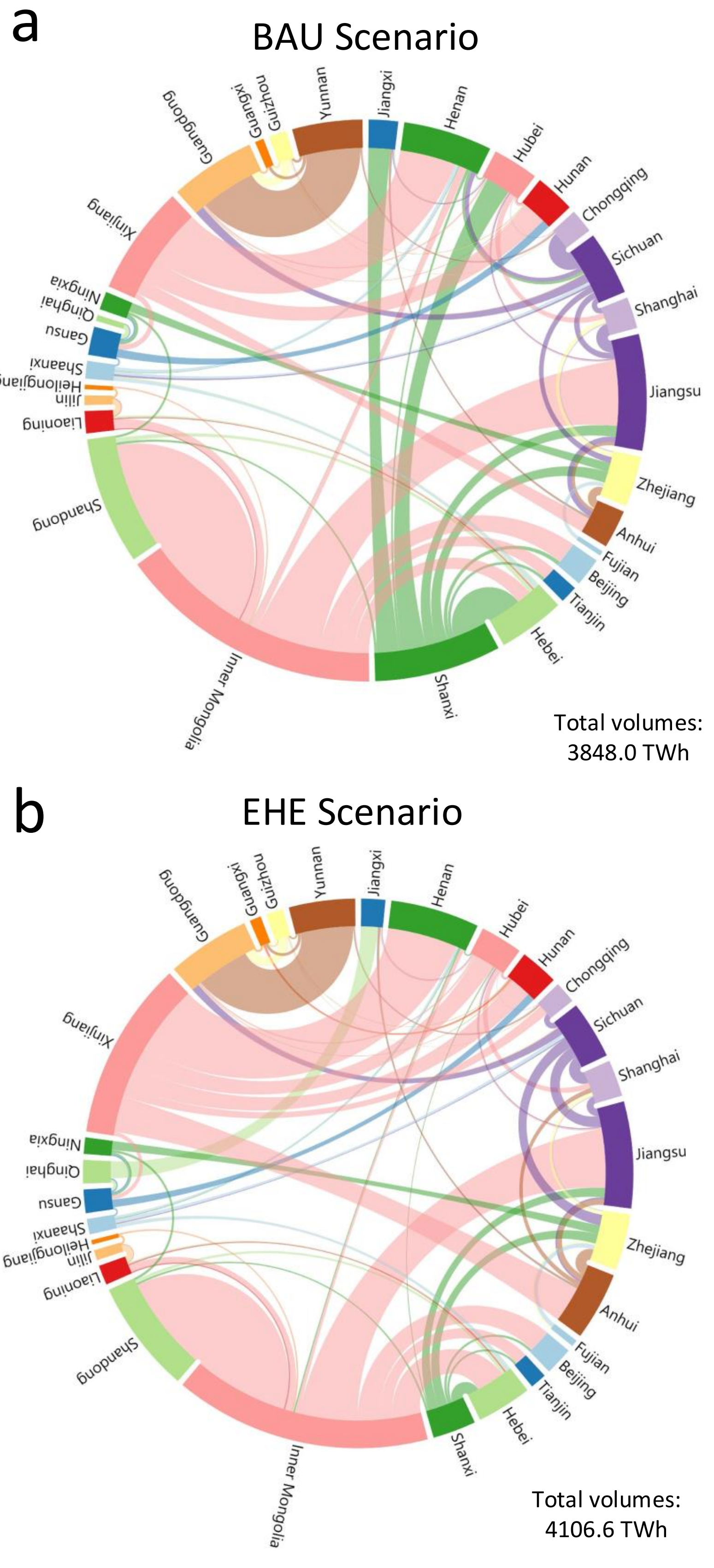

Figure S3. Inter-provincial electricity transmission volumes in 2035, Related to Figure 3. The subfigures show the total electricity transmission in the BAU scenario (a) and EHE scenario (b), respectively.

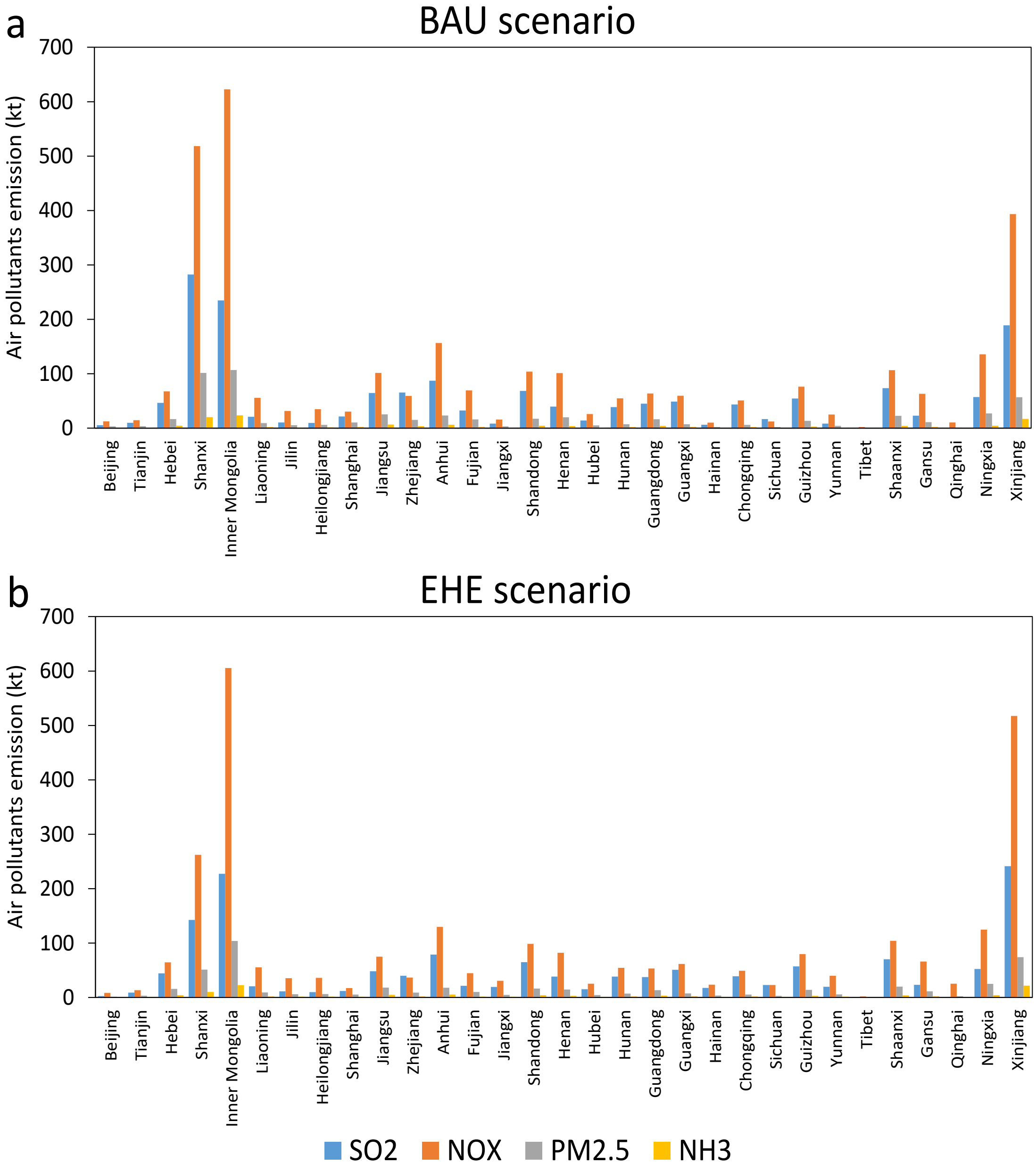

Figure S4. Provincial air pollutants emissions in 2035 under each scenario, Related to Figure 4.

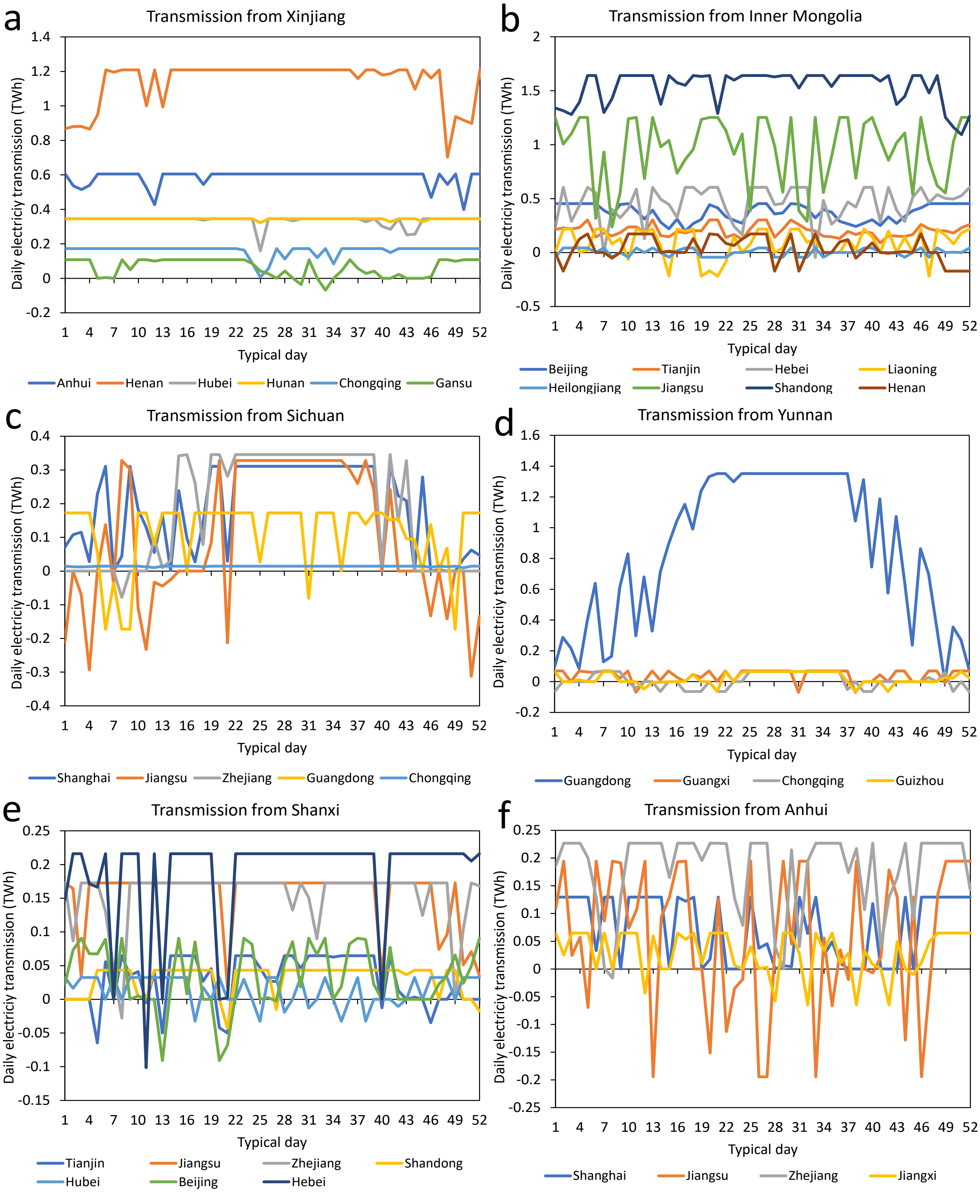

Figure S5. Daily electricity transmission volumes of some major electricity exporting provinces in the EHE scenario, Related to Figure 4.

The subfigures show the electricity transmission from Xinjiang (a), Inner Mongolia (b), Sichuan (c), Yunnan (d), Shanxi (e), and Anhui (f) to others, respectively.

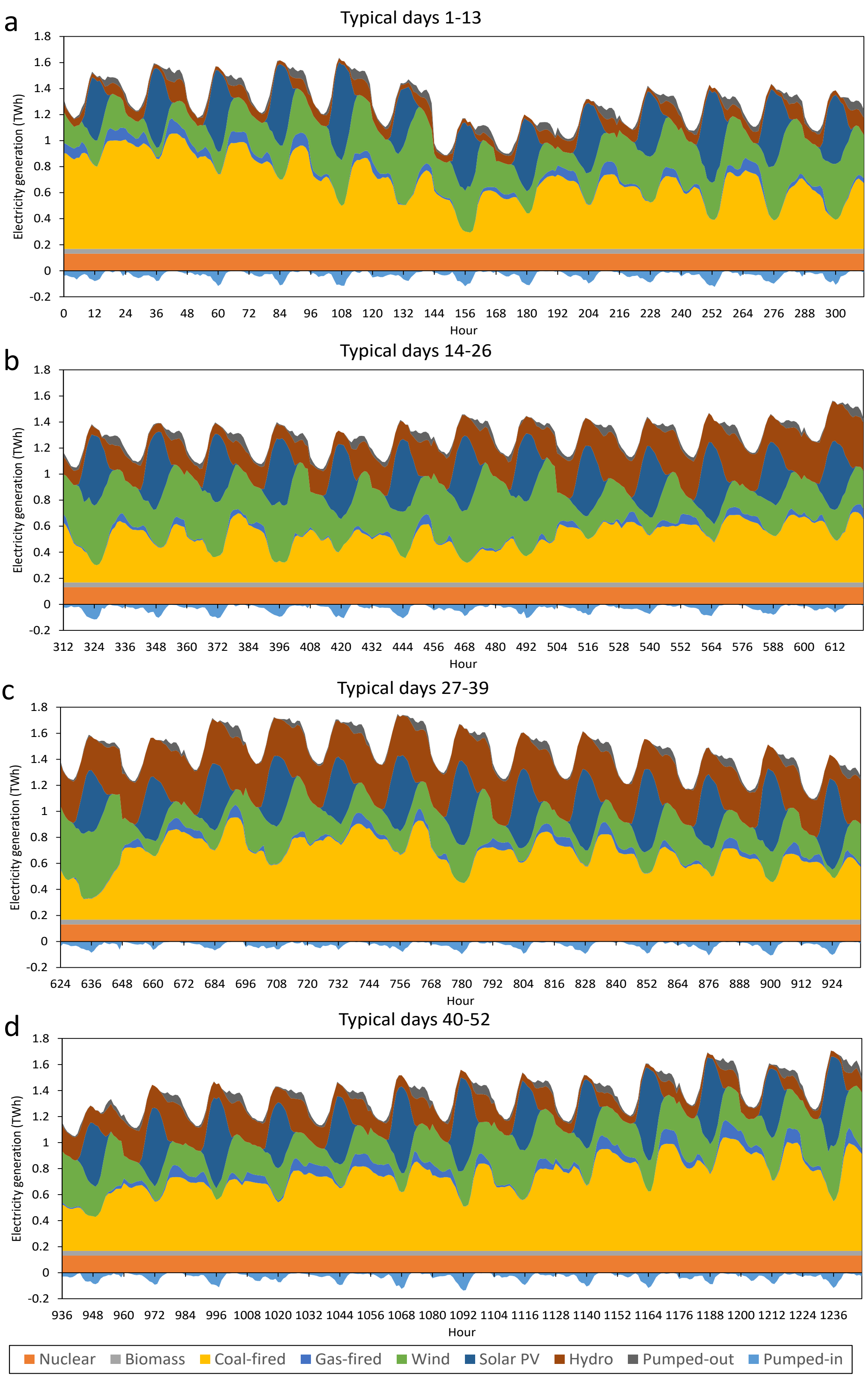

Figure S6. Hourly power dispatch at the national level in EHE scenario, Related to Figure 4. The subfigures show the electricity generation in typical days 1-13 (a), 14-26 (b), 27-39 (c), 40-52 (d), respectively. The order of typical days corresponds to the order of weeks. For example, typical day 27 is chosen during the 27th week of the year, which is usually summer in the Northern Hemisphere.

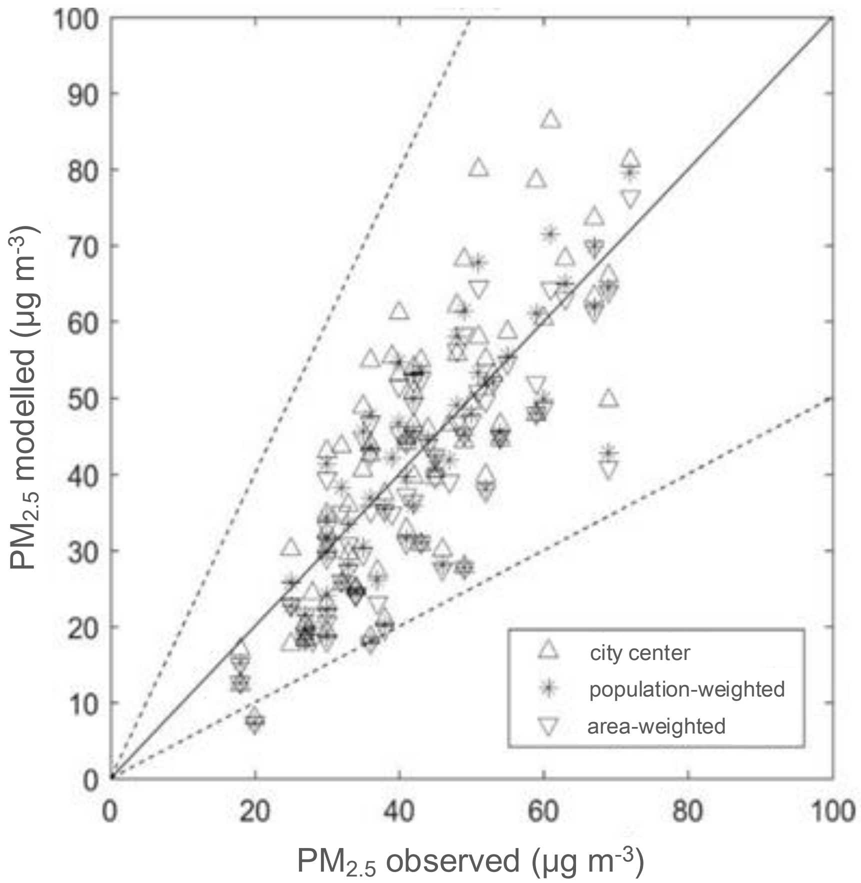

Figure S7. The validation plot between observed and modeled concentration of ambient $PM_{2.5}$ in 2018, Related to STAR Method.

The observations come from air quality reports from 74 major cities announced by the Ministry of Environmental Protection. Population-weighted concentrations are used in this study.

Supplemental Tables

Table S1. Total air pollutant emissions reduction from electricity transfers in each scenario, Related to Figure 4.

Scenario	External benefits (Billion CNY)	SO_2 (kt)	NO_X (kt)	$PM_{2.5}$ (kt)	NH_3 (kt)
BAU	73.403	31.94	-215.18	-17.11	-0.86
EHE	82.260	96.99	-133.95	-23.63	-2.62

Table S2. Model $PM_{2.5}$ concentration and total years of life lost, Related to STAR Method.

Province	Modeled $PM_{2.5}$ concentration ($\mu g\ m^{-3}$)	Total years of life lost (thousand years)	Concentration shares of power sector (%)
Beijing	55.20	360.76	2.18
Tianjin	54.85	260.69	4.16
Hebei	58.69	1293.01	3.82
Shanxi	43.36	566.19	6.39
Inner Mongolia	23.88	278.92	3.34
Liaoning	31.41	567.07	4.85
Jilin	21.55	276.94	3.31
Heilongjiang	19.69	360.94	3.45
Shanghai	46.37	379.72	6.85
Jiangsu	47.04	1268.56	5.50
Zhejiang	32.52	760.55	5.88
Anhui	48.85	1011.61	6.44
Fujian	20.34	386.60	6.12
Jiangxi	26.44	546.57	4.87
Shandong	48.83	1606.88	6.29
Henan	57.95	1636.50	5.68
Hubei	46.70	929.56	5.18
Hunan	34.59	944.61	3.81
Guangdong	25.56	1305.96	5.76
Guangxi	19.92	475.51	3.83
Hainan	12.18	56.02	4.93
Chongqing	38.17	445.86	3.98
Sichuan	41.39	1244.50	2.69
Guizhou	21.33	365.95	3.47
Yunnan	12.96	313.56	1.26
Tibet	2.13	0.00	0.02
Shaanxi	47.51	611.28	5.99
Gansu	27.27	316.08	3.16
Qinghai	21.27	61.17	3.79
Ningxia	30.14	87.46	7.43
Xinjiang	41.80	372.71	3.72

Table S3. Existing power generation capacity in 2035 (GW), Related to STAR Method.

Province	Coal	Gas	Nuclear	Hydro	Biomass	Wind	Solar	Pumped
Beijing	0.85	9.85	0	0.18	0.2	0.1	0.4	0.8
Tianjin	8.37	3.17	0	0	0.06	0.44	1.28	0
Hebei	33.29	0.01	0	0.55	0.72	10.16	12.34	1.27
Shanxi	42.97	2.17	0	1.03	0.32	9.89	8.64	1.2
Inner Mongolia	59.63	0.11	0	1.22	0.19	18	9.45	1.2
Liaoning	19.21	0.02	4.48	1.79	0.15	4.38	3.02	1.2
Jilin	13.74	0.005	0	3.55	0.61	2.81	2.65	0.3
Heilongjiang	12.42	0.05	0	1.04	0.97	4.07	2.15	0
Shanghai	11.91	3.14	0	0	0.27	0.58	0.89	0
Jiangsu	45.64	11.87	4.37	0.05	1.42	7.01	13.32	2.6
Zhejiang	33.36	8.84	9.08	7.03	1.1	1.26	11.38	4.58
Anhui	49.29	0.14	0	1.44	1.56	2.26	11.18	1.68
Fujian	20.06	2.59	8.71	12.02	0.34	2.42	1.48	1.2
Jiangxi	15.27	0.1	0	5.07	0.2	2.17	5.36	1.25
Shandong	60.06	0.05	1.25	0.08	2.52	9.64	13.61	1
Henan	52.93	1.09	0	2.69	0.57	4.63	9.91	1.32
Hubei	18.83	0.94	0	35.48	0.75	3.27	5.1	1.27
Hunan	14.13	0.04	0	14.78	0.67	3.44	2.92	1.2
Guangdong	42.92	8.56	13.3	8.48	0.88	3	5.27	7.28
Guangxi	13.92	0.35	2.17	16.77	1.12	2.08	1.24	0
Hainan	3.52	0.19	1.3	0.94	0.28	0.09	1.36	0.6
Chongqing	9.37	1.12	0	7.56	0.26	0.45	0.43	0
Sichuan	9.98	0.32	0	78.24	0.47	2.53	1.81	0
Guizhou	25.96	0	0	22.12	0.15	3.86	1.78	0
Yunnan	8.31	0.01	0	66.49	0.27	8.57	3.43	0
Tibet	0	0	0	1.51	0.02	0.01	0.02	0.09
Shaanxi	29.18	0.11	0	3.85	0.06	4.05	7.16	0
Gansu	16.44	0.04	0	9.27	0.1	10.07	8.28	0
Qinghai	3.75	0.004	0	11.92	0.004	2.67	9.56	0
Ningxia	22.83	0.22	0	0.43	0.06	9.35	8.14	0
Xinjiang	44.5	0.53	0	7.02	0.02	18.01	9.91	0

Note： These capacities don’t include newly built capacities between 2018 and 2035, which are the results of optimization by this study.

Table S4. Existing power transmission capacity in 2035, Related to STAR Method.

Ultra-high voltage line	Capacity (GW)	High voltage line	Capacity (GW)
Xinjiang – Anhui	12	Xinjiang – Gansu	5
Xinjiang – Henan	8	Ningxia – Gansu	7
Ningxia – Zhejiang	8	Qinghai – Gansu	5
Gansu – Hunan	8	Inner Mongolia – Beijing	9
Yunnan – Guangdong	15	Shanxi – Beijing	4.2
Sichuan – Shanghai	6.4	Inner Mongolia – Hebei	7
Sichuan – Jiangsu	7.2	Shanxi – Hebei	7
Sichuan – Zhejiang	8	Liaoning – Hebei	4.9
Inner Mongolia – Shandong	20	Inner Mongolia – Liaoning	10.1
Inner Mongolia – Jiangsu	10	Inner Mongolia – Heilongjiang	2
Shanxi – Jiangsu	8	Jilin – Liaoning	3.2
Jiangsu – Shanghai	6	Heilongjiang – Jilin	2.4
Zhejiang – Shanghai	6	Anhui – Jiangsu	3
Anhui – Jiangsu	6	Anhui – Zhejiang	4.5
Anhui – Zhejiang	6	Hubei – Shanghai	7.18
Fujian – Zhejiang	6	Hubei – Jiangsu	3
Inner Mongolia – Tianjin	3	Hubei – Jiangxi	2.4
Shanxi – Tianjin	3	Hebei – Shandong	6
Shaanxi – Hebei	4	Hubei – Hunan	3
Shanxi – Shandong	2	Hubei – Guangdong	3
Shanxi – Hubei	1.5	Hubei – Chongqing	2
Hubei – Henan	1.5	Sichuan – Chongqing	5
		Guizhou – Hunan	1.4
		Guizhou – Guangdong	10.8
		Guizhou – Chongqing	1
		Yunnan – Guangdong	7.6
		Yunnan – Guangxi	3.2
		Gansu – Shaanxi	2.6
		Ningxia – Shandong	4
		Sichuan – Shaanxi	3
		Hunan – Guangdong	0.6
		Shaanxi – Henan	1.1

Note： These capacities don’t include newly built capacities between 2018 and 2035, which are the results of optimization by this study.

Table S5. Electricity demand projections in 2035 (GWh), Related to STAR Method.

Province	Average hourly demand	Maximum hourly demand	Minimum hourly demand
Beijing	19.35	31.99	9.75
Tianjin	14.13	21.51	8.84
Hebei	60.39	82	41.18
Shanxi	35.42	46.57	28.02
Inner Mongolia	56.45	66.2	49.01
Liaoning	39.62	48.52	32.95
Jilin	12.78	17.36	8.41
Heilongjiang	16.44	22.49	11.27
Shanghai	25.25	39.57	15.74
Jiangsu	101.15	143.67	55.43
Zhejiang	73.82	107.77	27.04
Anhui	33.75	54.73	20.6
Fujian	37.79	51.63	17.96
Jiangxi	24.77	35.62	13.51
Shandong	97.78	136.11	61.51
Henan	58.31	90.56	38.11
Hubei	36.67	57.66	21.63
Hunan	29.89	45.79	16.51
Guangdong	107.23	150.09	37.11
Guangxi	28.85	41.91	15.32
Hainan	5.26	7.47	2.76
Chongqing	19.24	33.86	10.32
Sichuan	43.54	66.35	25.76
Guizhou	24.51	40.87	14.45
Yunnan	29.41	38.31	18.31
Tibet	1.32	2.28	0.83
Shaanxi	31.33	44.66	20.7
Gansu	25.93	31.89	21.76
Qinghai	15.58	17.22	14.2
Ningxia	21.24	25	17.21
Xinjiang	51.55	58.31	44.76

Note： The electricity demand has excluded the losses within the power plant and the region power grid.
